# Microbial Community and Biochemical Dynamics of Biological Soil Crusts across a Gradient of Surface Coverage in the Central Mojave Desert

**DOI:** 10.3389/fmicb.2017.01974

**Published:** 2017-10-23

**Authors:** Rakesh Mogul, Parag Vaishampayan, Mina Bashir, Chris P. McKay, Keith Schubert, Rosalba Bornaccorsi, Ernesto Gomez, Sneha Tharayil, Geoffrey Payton, Juliana Capra, Jessica Andaya, Leonard Bacon, Emily Bargoma, David Black, Katie Boos, Michaela Brant, Michael Chabot, Danny Chau, Jessica Cisneros, Geoff Chu, Jane Curnutt, Jessica DiMizio, Christian Engelbrecht, Caroline Gott, Raechel Harnoto, Ruben Hovanesian, Shane Johnson, Britne Lavergne, Gabriel Martinez, Paul Mans, Ernesto Morales, Alex Oei, Gary Peplow, Ryan Piaget, Nicole Ponce, Eduardo Renteria, Veronica Rodriguez, Joseph Rodriguez, Monica Santander, Khamille Sarmiento, Allison Scheppelmann, Gavin Schroter, Devan Sexton, Jenin Stephenson, Kristin Symer, Tatiane Russo-Tait, Bill Weigel, Mary B. Wilhelm

**Affiliations:** ^1^Chemistry and Biochemistry Department, California State Polytechnic University, Pomona, Pomona, CA, United States; ^2^Science Team, NASA/CSU Spaceward Bound, Pomona, CA, United States; ^3^Blue Marble Space Institute of Science, Seattle, WA, United States; ^4^Biotechnology and Planetary Protection Group, Jet Propulsion Laboratory, California Institute of Technology, Pasadena, CA, United States; ^5^Division of Endocrinology and Diabetology, Medical University of Graz, Graz, Austria; ^6^Ames Research Center, National Aeronautics and Space Administration, Mountain View, CA, United States; ^7^Department of Computer Science, Baylor University, Waco, TX, United States; ^8^SETI Institute, Mountain View, CA, United States; ^9^Department of Computer Science, California State University, San Bernardino, San Bernardino, CA, United States; ^10^College of Education, University of Texas at Austin, Austin, TX, United States; ^11^Teacher Core, NASA/CSU Spaceward Bound, Pomona, CA, United States; ^12^Orchard Academies 2B: Arts and Media, Bell, CA, United States; ^13^Foothills Middle School, Arcadia, CA, United States; ^14^Research Cohorts, NASA/CSU Spaceward Bound, Pomona, CA, United States; ^15^Maple Hill High School, Castleton-on-Hudson, NY, United States; ^16^American Academy of Innovation, Jordan, UT, United States; ^17^Center for Excellence in STEM Education, California Polytechnic State University, San Luis Obispo, CA, United States; ^18^Center for Math and Science Education, San Francisco State University, San Francisco, CA, United States; ^19^Smiley Elementary School, Redlands, CA, United States

**Keywords:** biological soil crusts, surface coverage, bacterial diversity, biochemistry, vertical column, topsoil, subsurface, metals

## Abstract

In this study, we expand upon the biogeography of biological soil crusts (BSCs) and provide molecular insights into the microbial community and biochemical dynamics along the vertical BSC column structure, and across a transect of increasing BSC surface coverage in the central Mojave Desert, CA, United States. Next generation sequencing reveals a bacterial community profile that is distinct among BSCs in the southwestern United States. Distribution of major phyla in the BSC topsoils included Cyanobacteria (33 ± 8%), Proteobacteria (26 ± 6%), and Chloroflexi (12 ± 4%), with *Phormidium* being the numerically dominant genus. Furthermore, BSC subsurfaces contained Proteobacteria (23 ± 5%), Actinobacteria (20 ± 5%), and Chloroflexi (18 ± 3%), with an unidentified genus from Chloroflexi (AKIW781, order) being numerically dominant. Across the transect, changes in distribution at the phylum (*p* < 0.0439) and genus (*p* < 0.006) levels, including multiple biochemical and geochemical trends (*p* < 0.05), positively correlated with increasing BSC surface coverage. This included increases in (a) Chloroflexi abundance, (b) abundance and diversity of Cyanobacteria, (b) OTU-level diversity in the topsoil, (c) OTU-level differentiation between the topsoil and subsurface, (d) intracellular ATP abundances and catalase activities, and (e) enrichments in clay, silt, and varying elements, including S, Mn, Co, As, and Pb, in the BSC topsoils. In sum, these studies suggest that BSCs from regions of differing surface coverage represent early successional stages, which exhibit increasing bacterial diversity, metabolic activities, and capacity to restructure the soil. Further, these trends suggest that BSC successional maturation and colonization across the transect are inhibited by metals/metalloids such as B, Ca, Ti, Mn, Co, Ni, Mo, and Pb.

## Introduction

**FIGURE 1:**
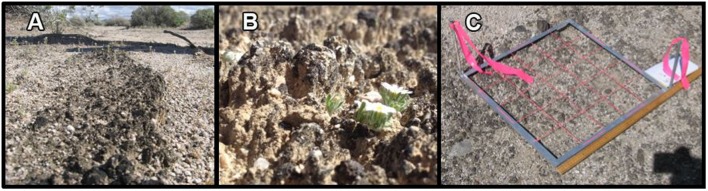
Images of black-crusted biological soils crusts (BSCs) in the western section of the Mojave National Preserve showing **(A)** example surface coverage at the high-density site, **(B)** pinnacled structure of the BSCs, and **(C)** the grid used for measuring BSC surface coverage.

Biological soil crusts (BSCs) play pivotal roles in the stability of dryland ecosystems (Belnap and Lange, 2003; Belnap, 2006) and significantly contribute to the global cycling of nitrogen (Elbert et al., 2012). Compositionally, BSCs are polyextremotolerant microbial topsoil communities (**Figures 1A,B**), comprised of bacteria, archaea, fungi, lichen, mosses, and diatoms, which are embedded together within a matrix of exopolysaccharides and soil particles to yield a surface crust. BSCs are common to arid and semi-arid environments and found in diverse geographies ranging from the Arctic circle (Breen and Lévesque, 2008; Steven et al., 2013) to the Namib desert in Africa (Büdel et al., 2009). Global distribution estimates suggest that desert and semi-arid ecosystems comprise ∼60% (Belnap, 1995; Elbert et al., 2012) of total surface land of the Earth, where averaged BSC coverages in these areas are estimated to be 40–50% (Garcia-Pichel et al., 2003; Elbert et al., 2012). Given these distributions, BSCs are unsurprisingly tolerant toward multiple environmental conditions including wide temperature fluctuations (∼-4 to 50°C), low-humidity, and exposure to ultraviolet radiation.

On a global scale, it is estimated that ∼30% of the total biological nitrogen fixation arises from cryptogamic ground covers, which include bryophytes, lichen carpets, rock crusts, and the differing successional stages of BSCs (Elbert et al., 2012). However, among the cryptogamic plant and ground covers of the Earth, the largest fluxes in nitrogen fixation are found in desert areas (in Africa, Asia, North America, South America, and Australia) (Elbert et al., 2012) where cyanobacterial crusts presumably dominate the cryptogamic ground cover. Moreover, at local scales, the metabolic and physical attributes of BSCs, including the carbon cycling (Belnap and Lange, 2003), water retention (Belnap, 2006), and dust accretion properties (Williams et al., 2013), significantly contribute to the hydrology, erosion stability, and fertility of arid soils. As a consequence, the measurement of phylogenetic composition, metabolic activities, and spatial distribution of BSCs potentially serve as important parameters for climate change and soil studies; where these metrics may function as biological indicators for alterations in soil nutrient cycling stemming from broad temperature and precipitation changes.

**FIGURE 2:**
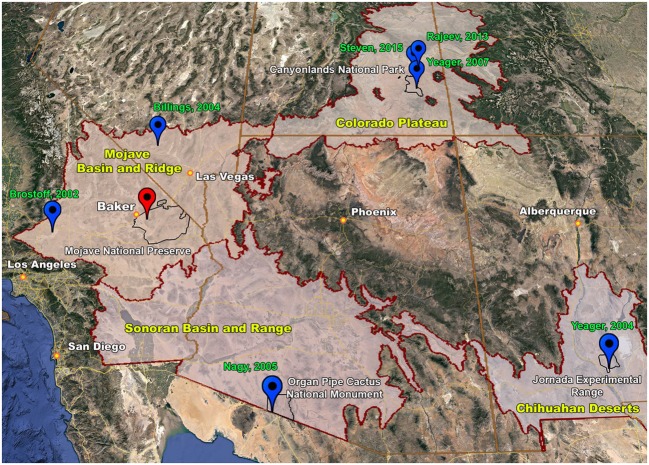
Biogeography of selected biological soils crusts (BSCs) in the southwestern United States; map shows the location (red icon) of BSCs characterized in this report (high-density site), the locations (blue icons) of other phylogenetically characaterized BSCs in the region (Author, year), the boundaries (maroon lines) of ecoregions defined by the Environmental Protection Agency (e.g., Mojave Basin and Ridge, Colorado Plateau, Sonoran Basin and Ridge, and Chihuahuan Deserts), and the boundaries (black lines) of associated national/federal lands; digital map was prepared using Google Earth Pro (7.3.0.3827, 64-bit), where GPS coordinates of the phylogenetically characaterized BSCs (blue icons) were obtained from the respective literature reports, boundary geographic data were downloaded from https://geodata.epa.gov/ https://catalog.data.gov https://jornada.nmsu.edu/ and all labels, and anotations added using Adobe Photoshop CC (2017.0.1 Release).

For the southwestern United States, several climate models predict increases in aridity, monsoonal rainfall, temperature, and CO_2_ levels, which together are expected to increase primary production in BSCs (Seager et al., 2007; Ustin et al., 2009). Geographically, the southwestern United States encompasses the Great Basin, Chihuahuan, Sonoran, and Mojave Deserts, which together surround and border the urban centers of Las Vegas and southern California. To date, however, the majority of in-depth phylogenetic interrogations on BSCs within this region (**Figure 2**) have focused on lower population areas (relative to southern California) including the Colorado Plateau in Utah, Sonoran Desert in Arizona, and Chihuahuan Desert in New Mexico (Bowker et al., 2002; Redfield et al., 2002; Yeager et al., 2004, 2007; Nagy et al., 2005; Soule et al., 2009). Across these studies, phylogenetic analyses of BSCs from the Colorado Plateau (Redfield et al., 2002; Steven et al., 2015) and Sonoran Desert (Nagy et al., 2005) reveal similar bacterial compositions. For instance, analysis of 16S rRNA amplicons (**Table 1**) show that Cyanobacteria are the dominant phyla, representing ∼38–55% of the total sequences, followed by Actinobacteria and Proteobacteria present at ∼12–16% (Nagy et al., 2005). Further, among the Cyanobacteria, both non-cultivation and cultivation-based studies indicate that *Microcoleus vaginatus* (Colorado Plateau) and *Microcoleus steenstrupii* (Sonoran Desert) are the more abundant species (Redfield et al., 2002; Nagy et al., 2005). Additionally, for BSCs from the Colorado Plateau (Yeager et al., 2007) and Chihuahuan Desert (Yeager et al., 2004), analyses of *nifH* sequences show that the *Nostocales* are the numerically dominant diazotrophic cyanobacterial order, with cultivation studies showing *Nostoc, Scytonema*, *Tolypothrix*, and *Spirirestis* as the best-represented genera among the diazotrophes.

**Table 1:** Bacterial compositions for BSCs from the southwestern United States; compositions of BSCs from the central Mojave Desert were measured using next generation sequencing (this study), while BSCs from the Sonoran Desert and Colorado Plateau were measured by Nagy et al. (2005) by Sanger sequencing of 16S rRNA amplicons (Nagy et al., 2005).

Central Mojave [next generation sequencing]				Sonoran Desert [16S rRNA amplicons^a^]		Colorado Plateau [16S rRNA amplicons^a^]	
	
Topsoil		Subsurface		Topsoil		Topsoil	
Cyanobacteria	33 ± 8%	Proteobacteria	23 ± 5%	Cyanobacteria	55%	Cyanobacteria	38%
Proteobacteria	26 ± 6%	Actinobacteria	20 ± 5%	Actinobacteria	15%	Proteobacteria	16%
Chloroflexi	12 ± 4%	Chloroflexi	18 ± 3%	Proteobacteria	14%	Actinobacteria	12%
Actinobacteria	9 ± 1%	Cyanobacteria	11 ± 10%	Acidobacteria	11%	Bacteroidetes	11%
Bacteroidetes	6 ± 2%	Acidobacteria	10 ± 2%	Bacteroidetes	0.9%	Firmicutes/Bacilli	5%
Acidobacteria	4 ± 1%	Bacteroidetes	6 ± 2%	Chloroflexi	0.7%	Thermomicrobiales	3%
FBP	3 ± 1%	FBP	4 ± 1%	Gemmatimonadetes	0.7%	Acidobacteria	3%
Gemmatimonadetes	3 ± 1%	Gemmatimonadetes	3 ± 1%	Deinococcus/Thermus	0.2%	Unaffiliated Alleles	13%
Armatimonadetes	1 ± 0.3%	Armatimonadetes	2 ± 0.5%	Unknown	2.7%		
Planctomycetes	0.6 ± 0.2%	Planctomycetes	1 ± 0.3%				
TM7	0.2 ± 0.1%	Nitrospirae	0.6 ± 0.2%				
		TM7	0.3 ± 0.2%				
		Chlorobi	0.2 ± 0.1%				
		Verrucomicrobia	0.2 ± 0.1%				


*^a^ As per Nagy et al. (2005), amplicons were prepared using universal bacterial primers [GM5F(GC) and 907R] and touchdown PCR.*

In this report, we expand upon the biogeography of cryptogamic covers and provide novel insights into the microbial community and biochemical dynamics of BSCs along the vertical column structure (topsoil and subsurface) and across a transect of increasing BSC surface coverage in the central Mojave Desert in southern California (or western section of the Mojave National Preserve). Across this transect, we reasoned that the noticeable changes in BSC surface coverage were likely associated with the early stages of BSC succession, which remains to be an understudied component of BSC development (Langhans et al., 2009; Zaady et al., 2010; Chamizo et al., 2012). Accordingly, and as described herein, we demonstrate through a suite of comparative molecular and biochemical analyses that BSC surface coverage correlates to several biological and geochemical trends, which together are consistent with the community and metabolic changes associated with early succession.

## Materials and Methods

### Materials

All centrifuge tubes, storage tubes, and mixing devices were obtained from VWR, ethanol (95%) and magnesium carbonate (MgCO_3_) were obtained from Fisher Scientific, and non-stabilized hydrogen peroxide (H_2_O_2_) was obtained from Sigma-Aldrich (and aliquoted and stored at -80°C upon receipt). All media and buffers were autoclaved at 121°C for 30 min or sterile filtered (0.22 μm); pure water (18 MΩ cm^-1^) was used throughout.

### Field Sites and Sample Collection

Samples were collected in March 2012, March and June 2014, and March 2016 in the Mojave National Preserve in southern California (**Figure 2**). Black-crusted BSC samples were obtained outside of Baker, CA, United States (off of Kelbaker Road) along a mild elevation transect (∼273–700 m over ∼21.5 km); where BSC surface coverages visibly increase as elevation rises, plateau at ∼700 m, and then decrease to very low surface coverages at the higher elevations. For this study, and as judged by visual observation, samples were collected at sites along the transect that had high (685 m; N 35 11.934, W 115 52.251), intermediate (450 m; N 35 15.313, W 115 57.429), and low (273 m; N 35 17.578, W 116 04.788) surface BSC coverages (or surface densities). At each site, BSC topsoils (top 1 cm of the black crust), BSC sub-surfaces (the following 1 cm of crust), and control soil samples (top 1 cm of adjacent soils containing no visible signs of black-crusted BSCs) were collected in triplicate using sterile spatula and stored in sterile 50 mL conical tubes. All samples were transported in a cooling box (at 4°C) within 2–4 h to the California State University Desert Studies Center (CSUDSC) in Zzyzx, CA, where several biochemical and initial nucleic acid analyses were performed using an on-site makeshift laboratory. Within 2 days of acquisition, samples were subjected to ATP abundance assays, chlorophyll abundances, and nucleic acid extractions (March 2012 samples). The extracted nucleic acids from all soil samples, as described below, were then transported to the Jet Propulsion Laboratory (JPL) at 4°C for further downstream characterizations, where quantitative polymerase chain reaction (qPCR) and 16S r RNA amplicon sequencing were performed within 3 weeks of acquisition. Unprocessed BSCs and soil samples were additionally transported to Cal Poly Pomona for further biochemical and geochemical analyses, which were performed within 4 weeks of acquisition. Samples collected during the March 2014 and 2016 field expeditions were collected, as described above, and analyzed for catalase specific activities within 2 days of acquisition (at the CSUDSC laboratory). Samples collected in June 2014 were stored in plastic storage bags at room temperature, and analyzed for elemental abundances and texture composition within 2 weeks of acquisition.

### Surface Density Measurements

Surface densities of the BSCs were estimated by pixel analysis of digital images obtained at five randomly selected areas within each sampling site (high, intermediate, and low-density surface coverage sites). The random locations (five locations per site) were normally distributed with zero mean and standard deviation of 10 m in both the north/south direction and the east/west direction, and were generated using SciLab’s rand function seeded by the time in seconds from January 1, 1970. Each area to be imaged was marked by two stakes to define the diagonal corners of a sampling grid, which was prepared using a square metal frame (20″ in interior on a side) that was divided into 25 squares (4″ on a side) using pink construction string (**Figure 1C**). Images of each grid were obtained using a Canon EOS 30D 8MP camera, which was fixed 3 ft from the ground using a tripod. Imaging settings included an ISO speed of ISO-100, F-stop of f/11, exposure time of 1/320 s, and focal length of 17 mm. Images were stored as an uncompressed tiff file (2336 × 3504 pixels) in 24-bit color (8-bit/channel) and processed using readily accessible graphics software (e.g., GIMP, Adobe Photoshop, and Paint). Images were analyzed separately by three to five different people and marked at the pixel level with contrasting colors for the BSCs (green) and metal frame (pink); where regions obscured by shadows (i.e., from marker flags and nearby objects) were marked (pink) and subsequently excluded from the analysis. Using a MATLAB script, pixels interior to the frame (interior to the pink pixels, and excluding all obscured areas) were enumerated by morphological filtering and used to calculate the percent of pixels marked as BSCs (green) among all pixels within the defined area. Surface densities (% area filled by the BSCs) were calculated for each processed image, and averages and standard deviations were calculated for each image (*n* = 3–5, per grid or image), across each sampling site (5 grids/site), and across clustered groups of site-specific data.

### Soil Analyses

Geochemical, texture, and elemental analyses were performed by the Brigham Young University Environmental Analytical Lab. Geochemical analyses included measurements of nitrate, ammonia-based nitrogen, total nitrogen, sulfate-based sulfur, total carbon, C:N ratios, electrical conductivity (EC, dS/M), % organic matter, and % moisture. Texture analyses included measurements of % sand, % clay, % silt, and overall classification. Elemental analyses included As, B, Ba, Cd, Co, Cr, Cu, Fe, Mn, Ni, Pb, S, Se, Sr, Ti, V, and Zn, as well as Ca, K, Mg, Na, P, and Si.

### ATP Abundances

Total and intracellular ATP were measured by bioluminescence using the CheckLite HS kit (Kikkoman), as described previously (Venkateswaran et al., 2003; La Duc et al., 2007). ATP abundances for each sample were measured by resuspending 1 g of sample (BSC or control soil) in 10 mL sterile phosphate buffered saline (PBS) in 50 mL sterile conical tubes (VWR^®^) with vigorous vortexing. Briefly, to determine total ATP (dead and viable microbes), equal volumes (100 μL) of homogenized sample aliquots and cell lysing detergent (benzalkonium chloride) were combined, and incubated at room temperature for 1 min before adding 100 μL of the luciferin–luciferase reagent. The sample was mixed, and the resulting bioluminescence was measured with a luminometer (Kikkoman). To determine intracellular ATP, 50 μL of an ATP-eliminating reagent (apyrase, adenosine deaminase) was added to 500 μL of the homogenized sample, incubated for 30 min to remove any extracellular ATP, and measured as described above. Sterile PBS was included in both assays as a negative control.

### Chlorophyll Abundances

Chlorophyll *a* (Chl *a*) abundances were measured using 2.0 g samples (top 1 cm of the black-crusted BSCs or control soils), where the samples were partially dried by heating at 40°C for 15 min followed by grinding using a mortar and pestle. Crushed samples were transferred to 15 mL conical tubes (VWR^®^), resuspended by vigorous vortexing (5–10 s) in 3–5 mL ethanol (neutralized by MgCO_3_) and heated at 80°C for 5 min. Samples were then agitated in an orbital shaker (VWR^®^) and cooled to room temperature over 30 min, whereupon the samples were clarified by centrifugation at 3000 × *g* for 3 min (Beckman Coulter Allegra 21R Centrifuge). Supernatants were collected and analyzed by absorption spectroscopy (240–800 nm) using a Shimadzu Bio-Spec 1601. Chlorophyll abundances were expressed as μg Chl *a*/g soil (Castle et al., 2011), and converted to μg Chl *a*/cm^2^ using BSC topsoil densities of 0.69 ± 0.16 (high), 0.42 ± 0.14 (intermediate), and 0.43 ± 0.13 (low) g/cm^3^, which were determined by measuring the mass of 1 mL of sample that had been collected in a 1.7 mL centrifuge tube (*n* = 5–8).

### Catalase Specific Activities

Catalase activities were measured by volume displacement (Bollag and Stotky, 1993; Nita et al., 2007) using a custom-built apparatus consisting of a sample reaction chamber, water reservoir, and collection chamber, with all components being sequentially connected with 10 mm tygon tubing (using 1-hole and 2-hole rubber stoppers for the reaction chamber and water reservoir, respectively). All samples (1.0 g BSCs and control soils) were ground for ∼1 min using a mortar and pestle, transferred to a 50 mL conical tube, and resuspended in 30 mL deionized water. The soil suspensions were constantly mixed using a magnetic stir bar, reactions were initiated by the addition of 320 mM H_2_O_2_ (final concentration), and the reaction chamber immediately sealed after the addition of H_2_O_2_. Reaction progress (at ∼22°C) was monitored by following the change in volume (or mass) of the liquid displaced from the water chamber due to the evolution of oxygen gas in the reaction chamber. Changes in displacement were measured every 30 s and reaction rates calculated by linear regression over 120 s using coefficients of determination of ≥0.99. Rates were converted to moles of oxygen evolved per second by the ideal gas law and the local barometric pressure (0.995 atm), temperature of the field laboratory (30°C), and corrections for water vapor to obtain the partial pressure of oxygen. Catalase specific activities were expressed as both Unit/g soil and μkat/g soil; where 1 Unit is equal to 1 μmole of hydrogen peroxide consumed per minute, and 1 μkat is equal to 1 μmole of hydrogen peroxide consumed per second.

### DNA Extractions

Each sample was homogenized by crushing BSC crusts and soil agglomerates using a sterile glass rod. Extractions of DNA from 10 g of subsample were performed using a PowerMax Soil DNA Isolation Kit (MoBio Lab, Carlsbad, CA, United States), by following the manufacturer’s protocol. The extraction protocol included both physical lysis, by vigorous vortexing with the PowerMax Bead suspension, and chemical lysis. Extracted DNA was stored at -20°C at the CSUDSC laboratory for ∼2 days, transported to JPL at 4°C, and stored at -80°C until further downstream analysis.

### Quantitative PCR Assay

Real-time qPCR assay was conducted in triplicate for each sample using a universal bacteria primer set targeting the 16S rRNA gene: 1369F (5′-CGG TGA ATACGT TCY CGG-3′), and modified 1492R (5′-GGW TAC CTTGTT ACG ACT T-3′) (Kwan et al., 2011). Reactions were performed on a Bio-Rad CFX-9600 thermocycler (Hercules, CA, United States) and PCR amplicon standards (16S rRNA gene of 10^8^–10^2^ gene copies/μL) were prepared from *Escherichia coli*. Each 25 μL reaction mixture consisted of 12.5 μL of Bio-Rad 2X iQ SYBR Green Supermix, 10.5 μL of UltraPure water (Gibco), 0.5 μL of forward primer 1369F (10 μM), 0.5 μL of reverse primer 1492R (10 μM), and 1 μL of DNA template. Purified standards and UltraPure Gibco water no-template controls were included in all qPCR runs. Thermal cycling parameters for universal 16S rDNA qPCR were as follows: incubation at 95°C for 3 min to achieve initial denaturation, followed by 40 cycles of 10 s at 95°C to denature, ramp-down to 55°C for primer annealing, and extension occurring through a 35 s ramp-up to 95°C.

### 454 Tag-Encoded Pyrosequencing

Universal Bacterial primers 28F (5′-GAG TTT GAT CNT GGC TCA G-3′) and 519R (5′-GTN TTA CNG CGG CKG CTG-3′) were used to amplify ∼500 bp fragments spanning the V1–V3 hypervariable regions of the bacterial 16S rRNA gene from each sample. This primer pair was tailored for bacterial tag-encoded FLX amplicon pyrosequencing (bTEFAP) by adding a fusion linker and a proprietary 12 base-pair barcode sequence at the 5′ end of the forward primer, and a biotin and fusion linker sequence at the 5′ end of the reverse primer (Dowd et al., 2008). A HotStarTaq Plus master mix kit (Qiagen, Valencia, CA, United States) was used to catalyze the PCR under the following thermal cycling conditions: initial denaturing at 95°C for 5 min, followed by 35 cycles of denaturing at 95°C for 30 s, annealing at 54°C for 40 s, and extension at 72°C for 1 min, finalized by a 10-min elongation at 72°C. Resulting PCR products were purified via Rapid Tip chemistry (Diffinity Genomics, Inc., West Henrietta, NY, United States), and were then pooled accordingly. Small fragments (<100 bp) were removed with Agencourt AMPure Beads in accordance with manufacturer’s instructions (Beckman Coulter, Brea, CA, United States).

In preparation for FLX-Titanium sequencing (Roche, Nutley, NJ, United States), resulting PCR amplicon fragment size and concentration were accurately measured with DNA 1000 chips using a Bioanalyzer 2100 automated electrophoresis station (Agilent, Santa Clara, CA, United States) and a TBS-380 Fluorometer (Turner Biosystems, Sunnyvale, CA, United States). The total volume of DNA products used for subsequent emulsion PCR was 2 μl for strong positives (>10 ng/μl), 5 μl for weak positives (5–10 ng/μl), and 20 μl for samples that failed to yield PCR products (<5 ng/μl). This normalization step helped to ensure minimal bias favoring downstream amplification from initially strong PCR products. Approximately 9.6 × 10^6^ molecules of ∼600 bp double-stranded DNA were combined with 9.6 × 10^6^ DNA capture beads, and then subjected to emulsion PCR conditions. Following recovery and enrichment, bead-attached DNA molecules were denatured with NaOH and sequencing primers were annealed. A 454 pyrosequencing run was performed on a GS PicoTiterPlate (PTP) using the Genome Sequencer FLX System in accordance with manufacturer’s instructions (Roche, Nutley, NJ, United States). Twenty-four to thirty tagged samples were applied to each quarter region of the PTP. All bTEFAP procedures were performed at the Research and Testing Laboratory (Lubbock, TX, United States) in accordance with well-established protocols (Dowd et al., 2008).

### Bioinformatics Analyses

Raw reads were processed with the open-source software mothur v.1.36.1 (Schloss et al., 2009) using the Schloss 454 standard operating procedure (October 2015^1^). In more detail, reads were denoized using PyroNoise (Quince et al., 2009) and aligned to the SILVA database v.119 (Pruesse et al., 2007; Quast et al., 2015). Pyrosequencing errors were reduced with pre-cluster allowing two differences and chimeras were identified with UCHIME (Edgar et al., 2011) and excluded. Non-bacterial sequences were identified by classification using mothur’s implementation of the ribosomal database project (RDP) classifier II and were excluded. After removal of non-bacterial sequences, remaining sequences were degapped, deuniqued, split into individual FASTA files per sample, and passed on to QIIME 1.9.1 (Caporaso et al., 2010). Subsequently, QIIME labels were added to each sequence and the resulting file was used for open reference operational taxonomic unit (OTU) picking using SortMeRNA (Kopylova et al., 2012) and for OTU picking against the Greengenes 13.8 database (DeSantis et al., 2006) using SUMACLUST (Mercier et al., 2013). An OTU was defined as a cluster of sequences with a similarity of 97% or more. The total sequences, ranging from ∼1650 to ∼4300, were minimized by exclusion of singletons and rarefied to 1660 OTU (unless otherwise specified) for all downstream statistical analyses. Alpha and beta-diversity were calculated and plotted in PhyloSeq (McMurdie and Holmes, 2013). Observed OTUs, Chao1, and parametric estimators were used to measure alpha-diversity; parametric estimators were calculated by fitting rarefaction data to a rectangular hyperbola using non-linear least squares regression, analogous to the Michaelis–Menten equation (IC_50_ Toolkit; ic50.tk), and solving for the theoretical maximum OTU value (or estimate of the asymptote), where the standard error of the regression (*S*) was calculated from the summed square of the residuals. Principal coordinates analyses were performed on Bray–Curtis dissimilarities, and Adonis was used to test for significant clustering between groups. Parametric *t*-tests were performed to compare the differences between groups, Benjamini–Hochberg false discovery rate was used to correct for multiple hypothesis testing, and canonical correspondence analysis (CCA) was used to extract gradients along multiple variables (R package Vegan). Raw sequence data were deposited at the Sequence Read Archive (SRA) under the study accession number SRP116344, BioProject PRJNA400492.

## Results

### BSC Surface Densities

**FIGURE 3:**
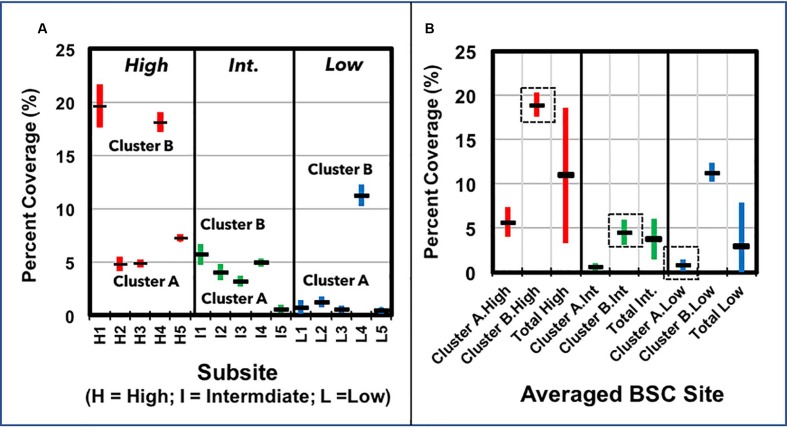
Plots of BSC surface coverage (%) across the transect, as measured by pixel analysis, showing **(A)** the surface densities calculated from each image (one image per grid, five grids per sampling site), with the respective clusters of values (within each site) grouped and marked as “Cluster A” and “Cluster B” (averaged surface densities are displayed as horizontal markers, and the standard deviation as vertical bars; *n* = 3–5, per image); and **(B)** the averaged BSC surface densities from Cluster A, Cluster B, and across each sampling site, where the representative values from each sampling site are marked by a dotted box (averaged surface densities are displayed as horizontal markers, and the standard deviation as vertical bars).

Surface densities of BSCs were calculated by pixel analysis of digital images (5 images/site), which were acquired at three sites across a mild elevation transect, where BSC surface densities visibly increase as elevation rises to ∼700 m. Sampling sites were broadly labeled as high (at 685 m), intermediate (at 450 m), and low (at 273 m) surface coverage areas, as judged by visual observation. Displayed in **Figure 3A** are the calculated surface densities from each image, where the standard deviation represents the variance from three to five independent analyses on each image (*n* = 3–5/image). At each site, the surface densities showed considerable variance, where the total averaged values at each site (**Figure 3B**) were 11 ± 7% (high-density), 4 ± 2% (intermediate-density), and 0.9 ± 0.4% (low-density), thus, suggesting substantial overlap in coverage between the sites. Accordingly, treatment of the imaging data as clusters of BSCs provided minimum and maximum coverages at each site; these clusters were suitably labeled as Cluster A and B (see **Figure 3B**). For the high-density site, clustering provided minimum and maximum surface coverages of 6 ± 1% (Cluster A) and 19 ± 1% (Cluster B). Likewise, at the intermediate-density site, the clustered surface coverages were 0.6 ± 0.1% and 5 ± 1%, whereas at the low-density site, the clustered surface coverages were 0.8 ± 0.4% and 11 ± 1%. Together, this provided a maximum surface coverage of 19 ± 1% at the high-density site (650 m) and predominant coverages of 5 ± 1% and 0.8 ± 0.4% at the intermediate (450 m) and low-density (273 m) sites, respectively.

### Transect and Soil Analysis

Geochemical and physical properties of the BSCs and control soil samples showed several correlations between surface density and C:N ratios, electrical conductivity, moisture content, and silt, sand, and clay content (**Table 2**). All BSC samples were classified as sandy loam, while adjacent control soils (non-black crusted BSCs) were loamy sand. Across the transect, increases in BSC surface density were associated with higher silt and clay content, and relatively lower sand content. In comparison, control samples showed no significant variations in silt, clay, or sand content across the transect. Among the BSCs, electrical conductivity and moisture content (at 1/3 bar) were higher at the high-density site, whereas C:N ratios decreased from ∼4, at the low-density site, to ∼1.7 at the high-density site.

**Table 2:** Selected physical and chemical properties of BSCs and control soils.

Sample	Texture	Sand	Clay	Silt	EC (S/m)	C:N Ratio	Moisture
High (H)	Sandy loam	59%	12%	29%	0.70	1.69	11.04%
Intermediate (I)	Sandy loam	67%	10%	23%	0.43	3.34	6.97%
Low (L)	Sandy loam	77%	8%	16%	0.55	4.08	6.69%
Control (H)	Loamy sand	83%	6%	11%	–	–	–
Control (I)	Loamy sand	87%	6%	7%	–	–	–
Control (L)	Loamy sand	86%	6%	9%	–	–	–


Elemental analysis (**Table 3**) of the BSC topsoils showed that increasing abundances of Co and Ti in the topsoil correlated with increasing BSC surface coverages. In contrast, increasing BSC surface coverages correlated with decreasing abundances of B, Ca, Cr, Ni, and Pb (when considering only the high and low-density sites for Ni); where these elements were highest in abundance in BSC topsoils at the low-density site. Similarly, for the control soils, the abundances of B, Ni, Mo, and Pb negatively correlated to surface coverage, and were the highest in abundance at the low-density site, including Ca, Ti, Mn, and Co when disregarding trends. Across the transect, no correlating trends were observed for Fe, Zn, Cd, Cu, Sr, Mg, P, Na, K, and Si. Comparison of the BSC topsoils against the respective controls showed evidence of enrichment in S, Ca, Ti, Cr, Mn, Co, As, Ba, and Pb (exceptions includes Ca and Co at the low-density site, and Cr at the intermediate-density site); where, among these elements, As showed the largest change in abundance with a ∼12-fold enrichment at the high-density site, with all other elements showing a 1.3- to 3-fold enrichment against the respective site-specific controls.

**Table 3:** Selected elemental compositions (ppm) of BSCs and adjacent control soils, including an indications of trends, where values in *bold* denote positive or negative correlations with BSC surface coverage, values in *italics* indicate enriched elemental abundances when compared to respective control soils, and underlined values denote high abundance elements at the low-density (BDL, below the detection limit).

Atomic number	5	16	20	22	24	25	27	28	33	42	56	82
**Sample (ppm)**	**B**	**S**	**Ca**	**Ti**	**Cr**	**Mn**	**Co**	**Ni**	**As**	**Mo**	**Ba**	**Pb**

High (H)	***35***	***88***	***7898***	***1210***	***33***	*249*	***6.5***	12	***1.8***	0.5	*88*	***9.4***
Intermediate (I)	***40***	*105*	***8943***	***1186***	**38**	*312*	***6.1***	10	*0.8*	0.6	*103*	***10.1***
Low (L)	***46***	***74***	**11926**	***891***	***42***	*256*	**5.9**	91	***0.8***	0.6	*81*	***13.4***
Control (H)	**32**	56	4409	761	27	198	4.5	**10**	0.1	**0.4**	68	**3.3**
Control (I)	**38**	31	4374	594	52	160	3.8	**74**	BDL	**0.7**	37	**4.3**
Control (L)	**41**	56	14003	817	40	242	58	**84**	BDL	**1.9**	66	**7.3**


### 16S rDNA Abundance

**FIGURE 4:**
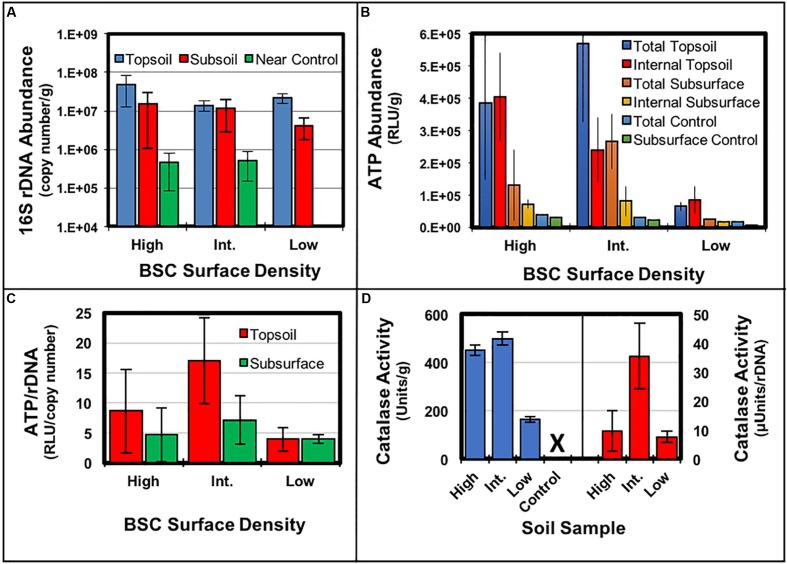
Comparison of molecular measurements across the transect and along the vertical BSC column: **(A)** 16S rDNA gene copy numbers, **(B)** ATP abundances in units of Relative Light Units (RLU) per gram of soil, **(C)** ATP specific activities in units of RLU per copy number of 16S rDNA, and **(D)** catalase specific activities expressed in both Units/g soil (blue, left axis) and μUnits/copy number of 16S rDNA (red, right axis); error bars represent the standard deviation (*n* ≥ 3) and ‘X’ denotes no measurable activity.

The 16S rDNA gene copy number abundances in the black crusted topsoil (top 1 cm), subsurface (following 1 cm) of the BSCs, and control soil from each site were estimated using qPCR assay (**Figure 4A**). Amplicon quantification across the transect provided averaged abundances of 2.8 × 10^7^ ± 1.7 × 10^7^ copy numbers/g for the BSC topsoils (or a range of 1.4 × 10^7^–4.7 × 10^7^ copy numbers/g), 1.0 × 10^7^ ± 0.56 × 10^7^ copy numbers/g sample for the BSC subsurfaces (or a range of 4.2 × 10^6^–1.5 × 10^7^ copy numbers/g), and 4.8 × 10^6^ ± 4.5 × 10^5^ copy numbers/g for the control soils (or a range of 4.5 × 10^6^–5.1 × 10^6^ copy numbers/g). Together, these values indicated that 16S rDNA copy numbers in the top black crust were equivalent across the transect, regardless of BSC surface density, while copy numbers from the high and low-density BSCs were ∼1-log higher than the control soils (*p* ≤ 0.05, *t*-test). Along the vertical column of the BSCs (topsoil vs. subsurface), the 16S rDNA abundances in the topsoil at the low-density site were ∼6-fold higher than the subsurface; however, no statistically significant differences were observed between the topsoils and subsurfaces at the high and intermediate-density sites.

### Metabolomic Measurements

Chlorophyll abundances, ATP abundances, and catalase specific activities were measured across the transect for BSCs and controls. Chlorophyll abundances were similar for all BSC samples (∼1.2 μg Chl *a*/g soil, ∼2.0 μg Chl *a*/cm^2^) and substantially lower in all controls soils (∼0.2 μg Chl *a*/g soil), thus suggesting that cyanobacterial content of the black-crusted BSCs were similar across the transect. The total ATP abundance trends (inclusive of both intracellular and extracellular ATP) were additionally similar for BSCs from the high and intermediate-density sites, with BSCs from the low-density site trending downward by ∼6-fold (**Figure 4B**). Across the transect, the abundances of intracellular ATP in the BSC topsoils (per gram) were ∼4.7-fold higher at the high-density site, when compared to the low-density site (*p* ≤ 0.05, *t*-test). In comparison to control soils, all BSC topsoils contained 10- to 20-fold higher intracellular ATP abundances. Comparison of the BSC subsurfaces showed that intracellular ATP increased ∼4.3-fold at the high-density site, when compared to the low-density site (*p* < 0.05, *t*-test). Across the transect, for all BSC topsoils, the total and intracellular ATP abundances were comparable, suggesting that intact cells were the major source of ATP. In contrast, in the BSC subsurfaces (at the intermediate and low-density sites), the total ATP abundances were ∼1.5- and 3.3-fold higher than the intracellular ATP, respectively (*p* < 0.05, *t*-test); thereby potentially revealing the presence of lysed cells and/or extracellular ATP-dependent processes in the subsurface (the high-density site did not show the same trend presumably due to high standard deviation in the total ATP abundances). Comparison along the vertical column showed that intracellular ATP abundances were ∼6 and 3-fold higher in the BSC topsoils at the high and intermediate-density sites, when compared to the respective subsurface samples (*p* < 0.05, *t*-test). At the low-density site, intracellular ATP abundances were equivalent in BSC topsoil and subsurface.

For comparative purposes (**Figure 4C**), the intracellular ATP abundances were also expressed as ATP/rDNA copy number (RLU/copy number); where the 16S rDNA copy numbers served as relative measures of bioburden, which were similar across the transect. Comparison across the intermediate and low-density sites showed a ∼4.3-fold higher ATP/rDNA ratio in BSC topsoils (2σ difference) and ∼1.8-fold higher ratio in the BSC subsurface (1σ difference) at the intermediate-density site; however, no inferences could be made from the high-density site due to high propagated error (**Figure 4C**). Along the vertical BSC column structure, the topsoils at the intermediate site contained a ∼2.4-fold higher ATP/rDNA ratio, when compared to the subsoil (1σ difference), while no differences were observed at the low-density site.

Catalase specific activities from the BSC topsoils (**Figure 4D**) were measured along the transect by volume displacement, as potassium permanganate titrations provided irreproducible and unusable data (presumably due to the high carbon content of the BSCs). As such, volume displacement provided the specific activities of 450 ± 23 Units/g (7.5 ± 0.4 μkat/g), 500 ± 26 Units/g (8.3 ± 0.4 μkat/g), and 160 ± 11 Units/g (2.7 ± 0.2 μkat/g) for BSC topsoils from the high, intermediate, and low-density sites, respectively. In context, both the high and intermediate-density BSCs had ∼3-fold higher specific activities when compared to the low-density BSCs (*p* < 0.05, *t*-test); whereas the high and intermediate-density sites showed significant overlap (*p* > > 0.05), similar to the trends in total and internal ATP abundances. At all sites, no appreciable catalase activities were measured in the control samples. Measurements (completed in March 2016) were also performed along the vertical column of BSCs from the high-density site, which showed ∼10-fold higher catalase specific activities in the topsoils, when compared to the subsurfaces (data not shown). Lastly, the catalase specific activities were also expressed per rDNA copy number (μUnits/rDNA copy number), which (similar to the ATP measurements) revealed that BSC topsoils from the intermediate-density site contained ∼4.5-fold higher ratios of catalase activity when compared to the low-density site (2σ difference); for these experiments, no comparisons could be made for the high-density site due to high standard deviation.

### Microbial Diversity

Microbial diversity assessment and comparative community analysis of the BSCs were performed using a next generation sequencing approach. Averaging of the total sequences across the transect (**Table 4**) yielded 2205 ± 726 and 2595 ± 1094 sequences in the topsoils and subsurfaces, respectively. Rarefaction analysis and hyperbolic regression (*S* = 4.0–8.4%) provided maximum theoretical OTU values of 811 ± 131 in the topsoils and 1080 ± 178 in the subsurfaces, as calculated by averaging of the regression parameters across the respective samples (**Supplementary Figure S1**). Comparison of these values to the measured OTUs (**Table 4**) indicated reasonable sequencing coverages of 67 ± 8% and 71 ± 9% for the BSC topsoils and subsurfaces, respectively. Further, comparisons of the maximum theoretical OTU values along the vertical BSC column (topsoil vs. subsurface) indicated greater diversity in the BSC subsurfaces (*p* = 0.0024, *t*-test); this assessment was additionally indicated in non-parametric testing (observed OTUs and Chao1) using Monte Carlo permutations (*p* = 0.003; **Supplementary Figure S2**).

**Table 4:** Comparison of total sequences and measured OTU values across the transect (OTU was defined as a group of sequences with a similarity of 97% or more).

	Total sequences		Total OTU	
		
Sample	Topsoil	Subsurface	Topsoil	Subsurface
High	2120 ± 91	3060 ± 1444	585 ± 25	867 ± 213
Intermediate	1866 ± 38	1996 ± 141	484 ± 15	622 ± 80
Low	2629 ± 1284	2729 ± 1340	557 ± 172	826 ± 238


**FIGURE 5:**
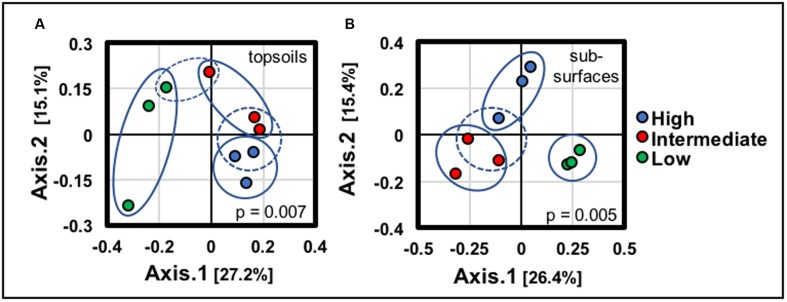
Environmental clustering across the transect using principle component analysis for **(A)** all BSC topsoils and **(B)** all BSC subsurfaces (blue ovals represent clustering; dotted blue ovals highlight overlaps in the clustering; signficance values for each analyses are provided in the plots).

Environmental clustering *via* ordination analyses (**Figure 5**) indicated that BSCs from the low-density site, both topsoil and subsurface, clustered separately (along axis 1) from the BSCs at the high and intermediate density sites (blue ovals in **Figure 5**), thereby supporting a difference in BSC community profiles across the transect (topsoils, *p* = 0.007; subsurfaces, *p* = 0.005). However (as indicated as dotted blue ovals in **Figure 5**), BSCs from the high and intermediate-density sites showed appreciable overlap along axis 1, but noticeable separation along axis 2, thereby suggesting the presence of similar communities at the high and intermediate-density sites. Significantly, this overlap between the high and intermediate-density sites was also observed in the total averaged surface densities, internal and total ATP abundances, and catalase specific activities. These similarities supported a phylogenetic and biochemical equivalence between the high and intermediate-density sites.

**FIGURE 6:**
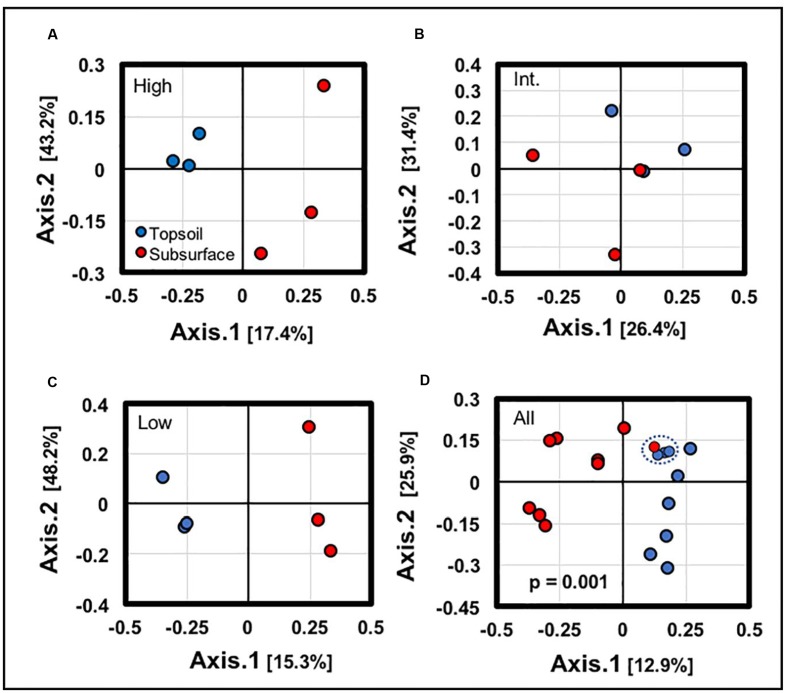
Independent ordination analyses of samples along the vertical column structure of BSCs from **(A)** the high-density site, **(B)** the intermediate-density site, **(C)** the low-density site, and **(D)** across all sites (dotted blue ovals highlight overlaps in the clustering between topsoil and subsurface; signficance values for each analyses are provided in the plots).

Statistical comparisons conducted along the vertical BSCs column supported the presence of distinct topsoil and subsurface community profiles. Ordination analyses (**Figures 6A–C**) conducted across the topsoils and subsurfaces at each site (low, intermediate, and high density sites) showed clear separation for BSCs from the high and low-density sites (along axis 1), with the intermediate-density site showing appreciable overlap (along axis 1). Similarly, ordination analyses conducted across all samples (**Figure 6D**) showed clear separation between the BSC topsoils and subsurfaces (*p* = 0.001). However, in this comparison (**Figure 6D**), a subset of samples showed minimal separation along axis 2 and appreciable overlap along axis 1 (blue dotted line); this subset included 1 subsoil sample from the intermediate-density site and 3 topsoil samples from the high-density site.

### Phylum-Level Analysis

**FIGURE 7:**
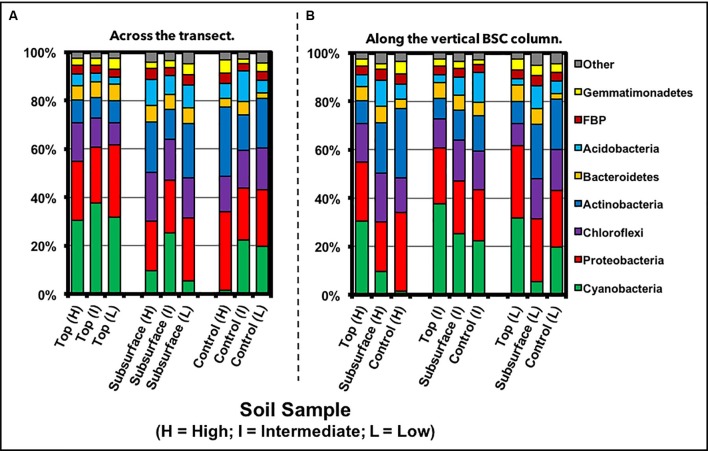
Phylum-level distribution of community members from samples **(A)** across the transect and **(B)** along the vertical column of the BSCs, as measured through next generation sequencing.

Phylum-level analysis across all BSCs, as displayed in **Figure 7** and listed in **Table 1**, showed that the total topsoil sequences were dominated by Cyanobacteria (33 ± 8%), Proteobacteria (26 ± 6%), Chloroflexi (12 ± 4%), Actinobacteria (9.1 ± 1.3%), and Bacteroidetes (6 ± 2%), with Acidobacteria, FBP [candidate division FBP (Lee et al., 2013)], Gemmatimonadetes, Armatimonadetes, Planctomycetes, and TM7 comprising the lower abundant members (<5%). To reveal deeper insights into phylogenetic differences across the transect, two-way comparisons were conducted across the high and low-density sites (with the assumption that the high and intermediate sites were statistically equivalent), where the Benjamini–Hochberg critical value included all possible comparisons (*p* < 0.0439, *t*-test, false discovery rate of 0.25). Accordingly, two-way analyses across the sampled BSC topsoils indicated that Chloroflexi and Verrucomicrobia abundances were different. For Chloroflexi, the relative sequence abundances were 16 ± 2% at the high-density site and significantly lower at 8.9 ± 0.6% at the low-density site. In contrast, sequences for Verrucomicrobia were undetectable in the high-density site and 0.091 ± 0.03% at the low-density site.

For the BSC subsurfaces, as displayed in **Figure 7** and listed in **Table 1**, phylum-level distribution showed considerable more diversity than the topsoils, with the higher abundant members including Proteobacteria (23 ± 5%), Actinobacteria (20 ± 5%), Chloroflexi (18 ± 3%), Acidobacteria (10 ± 2%), Bacteroidetes (6.5 ± 2.0%), and Cyanobacteria (11 ± 10%); in contrast, FBP, Gemmatimonadetes, Armatimonadetes, Planctomycetes, Nitrospirae, TM7, Chlorobi, and Verrucomicrobia represented the lower abundant members (<5%). Across the transect, two-way comparisons showed that Gemmatimonadetes and Fibrobacteres abundances in the BSC subsurfaces changed between the high and low-density sites. For Fibrobacteres, sequences were undetectable at the low-density site and increased to 0.23 ± 0.08% at the high-density site; whereas for Gemmatimonadetes, sequence abundances were 4.4 ± 0.4% at the low-density site and decreased to 2.5 ± 0.3% at the high-density site.

Specific differences along the vertical BSC column, or between the topsoil and subsurface, at the high and low-density sites were also obtained using two-way comparisons. At the high-density site, the topsoil was enriched in Cyanobacteria (∼3-fold higher), while the subsurface contained higher abundances of Acidobacteria (∼2-fold), Actinobacteria (∼2-fold higher), Nitrospirae (∼6-fold), and WPS-2 (undetectable in the topsoil). At the low-density site, cyanobacterial abundances were essentially equivalent along the vertical structure, while higher abundances of Acidobacteria (∼3-fold), Actinobacteria (∼2-fold higher), Chloroflexi (∼1.5-fold), Nitrospirae (∼47-fold), and TM7 (∼2-fold higher) were found in the subsurface.

### Genus-Level Diversity

**FIGURE 8:**
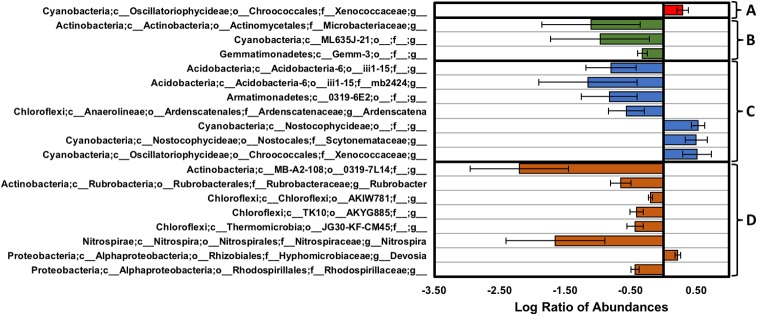
Changes in genus-level distribution expressed as the log of the ratio between **(A)** the BSC topsoils from the high- and low-density sites (red), **(B)** the BSC subsurfaces from the high- and low-density sites (green), **(C)** the BSC topsoils and subsurfaces from the high-density site (blue), and **(D)** the BSC topsoils and subsurfaces from the low-density site (orange); postive values along the *x*-axis indicate higher abundances in **(A,B)** samples from the high-density site or **(C,D)** the topsoils, while negative values, respectively, indicate lower abundances (error bars represent the propogated error).

Genus-level analyses, as summarized in **Figure 8**, showed several changes in abundance across the transect and along the vertical column, where comparisons across the high and low-density sites were performed using a Benjamini–Hochberg critical value that included all possible tests (*p* < 0.006, *t*-test, false discovery rate of 0.25). At the high-density site, sequences from the BSC topsoils were dominated by *Phormidium* (Cyanobacteria) and an unidentified genus from Chloroflexi (AKIW781, order), with both genera comprising ∼14% of the total sequences. Comparisons across the high and low-density sites (**Figure 8A**) showed that the BSC topsoils at the high-density site contained higher abundances of genera from the Cyanobacteria (∼2-fold enrichment to yield a ∼6% relative abundance for an unidentified genus from the *Xenococcaceae* family) and Proteobacteria (*Mesorhizobium* and an unidentified genus from the Burkholderiales order, which were both undetectable at the low-density site, and <1% at the high-density site).

For the BSC subsurfaces, sequences from the high-density site were dominated by an unidentified genus from Chloroflexi (AKIW781, order) and *Rubrobacter* (Actinobacteria), with both genera comprising ∼15% and 6.7% of the site-specific sequences, respectively. Comparison across the transect (**Figure 8B**) showed that BSC subsurfaces from the low-density site contained higher abundances of Actinobacteria (∼13-fold enrichment to ∼1.8% for an unidentified genus from the *Microbacteriaceae* family; and *Kribbella*, which was undetectable at the high-density site, and <1% at the low-density site), Cyanobacteria (∼9-fold enrichment to ∼2% for an unidentified genus from the ML635J-21 class), and Gemmatimonadetes (∼2-fold enrichment to ∼4% for an unidentified genus from the Gemm-3 class).

Comparisons along the vertical column (**Figure 8C**) showed that BSC topsoils at the high-density site contained higher abundances of genera from the Cyanobacteria (∼3-fold enrichments to ∼2.3, 1.3, and 5.8% for unidentified genera from the Nostocophycideae order, *Scytonemataceae* family, and *Xenococcaceae* family, respectively); while the subsurfaces contained higher abundances of Acidobacteria (∼6- and 14-fold enrichments to ∼1.9 and 2.1% for unidentified genera from the iii1-15 class and *f__mb2424* family, respectively), Armatimonadetes (∼7-fold enrichment to <1% for an unidentified genus from the 0319-6E2 class), Bacteroidetes (an unidentified genus from the *Sphingobacteriaceae* family, which was undetectable at the high-density site, and <1% at the low-density sites), Chloroflexi (∼4-fold enrichment to ∼8% for *Ardenscatena*), and Proteobacteria (an unidentified genus from the Burkholderiales order, which was also undetectable at the high-density site, and <1% at the low-density sites). In contrast, the BSC topsoils at the low-density site (**Figure 8D**) contained marginally higher abundances of the *Devosia* genus of the Proteobacteria (∼1.7-fold enrichment to <1%), while the subsurfaces contained higher abundances of Actinobacteria (∼4.5-fold enrichment to 8% for *Rubrobacter*, and ∼160-fold enrichment to 1.3% for an unidentified genus from the 0319-7L14 order), Chloroflexi (∼1.6-, 2.6-, and 2.7-fold enrichments to ∼11, 1, and 1% for unidentified genera from the AKIW781, AKYG885, JG30-KF-CM45 orders, respectively), Cyanobacteria (an unidentified genus from the SM1D11 order, which was undetectable in the topsoil), Nitrospirae (∼45-fold enrichment to ∼1% of *Nitrospira*), and Proteobacteria (∼2.7-fold enrichment to ∼1.4% for an unidentified genus from the *Rhodospirillaceae* family).

**FIGURE 9:**
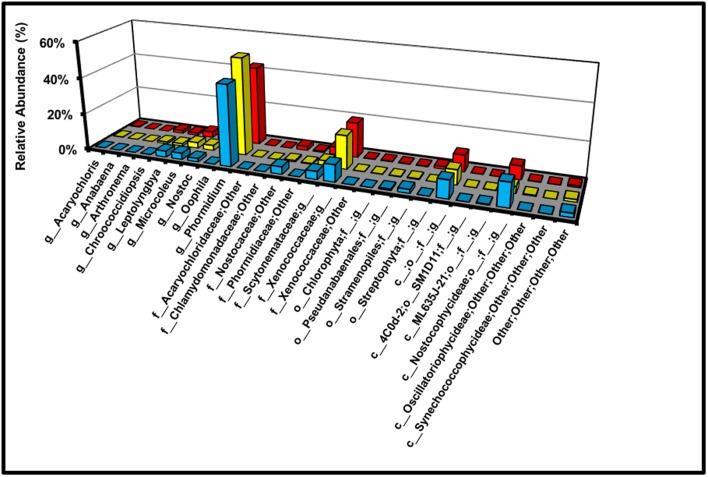
Relative abundance of cyanobacterial genera across the transect (g = genus; f = family; o = order; c = class).

For demonstrative purposes, the relative abundances of all cyanobacterial genera in the BSC topsoils are also displayed in **Figure 9**. Across the transect, and at all sites, the dominant cyanobacterial genus was *Phormidium* (47 ± 17%) with minor members including an unidentified genus from the *Xenococcaceae* family (19 ± 2%), a strain unclassified at the genus, family, order, and class levels (9 ± 2%), and an unidentified genus from the Nostocophycideae class (7 ± 1%). Notably, the relative percentages of *Microcoleus* and *Nostoc* were only between 1% and 3%. Interestingly, very low abundances of sequences associated with Chlorophyta (0.35%) and Streptophyta (1.5%) were measured at the high-density site, yet were undetectable at the low-density site (*p* ≤ 0.05, 1 sample *t*-test, 1-tailed); while tentative, these results hint at the presence of algae (or contaminating plant matter) at the high-density site.

### OTU-Level Diversity and Gradient Analysis

**FIGURE 10:**
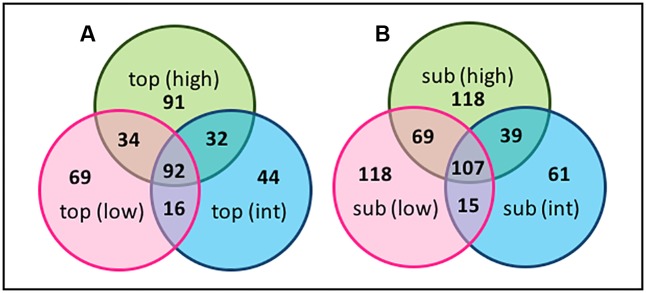
Venn diagrams of OTU-level diversity among the **(A)** the BSC topsoils and **(B)** BSC subsurfaces across the transect, where numbers represent the OTU values.

**FIGURE 11:**
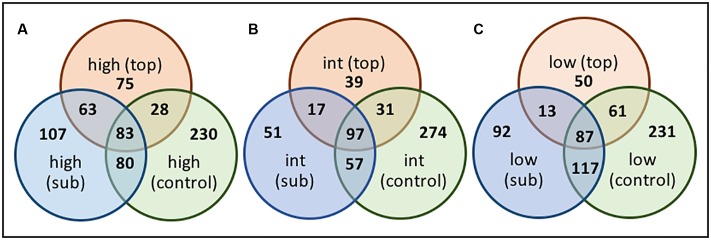
Venn diagrams of OTU-level diversity along the vertical column of BSCs from the **(A)** high-, **(B)** intermediate-, **(C)** and low-density sites, where numbers represent the OTU values.

Sequences were clustered at the OTU level and changes in the relative distribution of the OTUs across the transect, and along the vertical BSC column structure, visualized using Venn diagrams (**Figures 10**, **11**). Due to the short sequencing reads, species-level taxa were not assigned; instead, Venn diagrams were constructed using a similarly threshold of 97%, an 85% cutoff for calculation of the core population, and by plotting taxa that were present in at least 85% of all samples within the group^2^. As shown in **Figure 10A**, the number of OTUs specific to the topsoils increased from 69 to 91 when comparing the low- and high-density sites, indicating an overall increase in bacterial diversity across the transect. The topsoils were dominated by several OTUs from the *Phormidium* and several OTUs from the AKIW781 order of the Chloroflexi, and the core microbiome was represented by 92 OTU. In contrast, as shown in **Figure 10B**, the OTUs specific to the subsurfaces at the high and low-density sites remained constant at 118 OTU, suggesting a minimized change in diversity, with the core microbiome representing 107 OTU.

Comparisons along the vertical column (**Figure 11**) also supported greater diversification over the transect. For example, when comparing the low and high-density sites (**Figures 11A,C**), the number of topsoil-specific OTUs increased 50% (from 50 to 75 OTU), while the subsurface-specific OTUs increased ∼16% (from 92 to 107 OTU). Across all sites, the core microbiome between the topsoils and subsurfaces remained relatively constant at 89 ± 7 OTU. For these comparisons, the intermediate-density site (**Figure 11B**) provided limited correlations, presumably due to insufficient distinction from the high- and/or low-density sites. For comparative purposes, OTUs from the control soils (which were sampled once at each site, analyzed, and the sequences pooled) were also included in the analysis. As such, the total controls soils showed substantial (though consistent) diversity at 245 ± 25 OTU. Nevertheless, when comparing across the low and high-density sites, the overlapping populations between the control soils and BSC topsoils decreased by ∼55% (from 61 to 28 OTU) and subsurfaces by ∼32% (from 117 to 80 OTU). These changes supported an increase in OTU-level differentiation between the BSCs and control soils across the transect.

**FIGURE 12:**
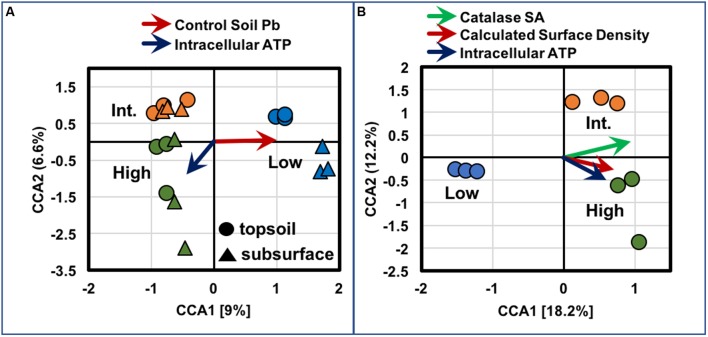
Plots of results from CCA for **(A)** BSC topsoils and subsurfaces and **(B)** BSC topsoils only, where tripilcate measures from each sampled site are shown in circles (topsoil) and triangles (subsurfaces), and color coded using the broad observed descriptors of high- (green), intermediate- (orange), and low-density sites (blue); arrows represent eigenvectors indicating direction/degree of correlation.

Gradient analyses on OTUs across the transect were performed by CCA. As displayed in **Figure 12A**, ordination analyses were conducted across the topsoils and subsurfaces from all sites (*n* = 3/sample) using intracellular ATP as a quantitative explanatory variable across all samples. For these analyses, the abundance of lead (Pb) in the control soils, from each specific site, was used as a categorical explanatory variable, since metal abundances were obtained from pooled samples of BSC topsoils from each site, along with the respective controls, and not measured in the subsurface samples. Hence, for these comparisons, Pb abundances effectively served as a proxy for any elements trending with BSC surface coverage. Under these assumptions, all topsoils and subsurfaces from the low-density site grouped together in the positive quadrants along the first axis, with the high- and intermediate-density samples clustering together in the negative quadrants along the first axis, and grouping separately and vertically along the second axis (15.6% total variance). These results were suggestive of distinct biochemical and bacterial community profiles for the BSCs across the transect, where increases in Pb (or B, Ni, and Mo) abundances correlated to BSCs from the low-density site, while increasing intracellular ATP samples correlated to BSCs from the high-density site (as inferred from the arrows or eigenvectors in **Figure 12A**). As displayed in **Figure 12B**, ordination analyses were also conducted solely on the BSC topsoils (*n* = 3/site) using the quantitative explanatory variables of calculated surface density, intracellular ATP, and catalase specific activity (Units/g); which were measured for each topsoil sample. Under these assumptions, the topsoils, respectively, grouped into differing quadrants, and all vectors clearly correlated with BSCs from the high- and intermediate-density sites (30.4% total variance). Again, these results were suggestive of distinct biochemical and bacterial community profiles for the BSCs across the transect, where increases in calculated surface density correlated to changes in taxa, increases in intracellular ATP, and increases in catalase specific activity.

## Discussion

Along the chosen geospatial transect, BSC surface coverages increased from 0.8 ± 0.4% to 19 ± 1%, as elevation increased from 273 m to 650 m. At each site, bacterial 16S rDNA copy numbers were essentially equivalent (2.8 × 10^7^ ± 1.7 × 10^7^ copy numbers/g, topsoil; 1.0 × 10^7^ ± 0.56 × 10^7^ copy numbers/g, subsurface), with the BSC topsoils containing ∼1-log higher abundances than control soils, which contained no visible black crust, yet quantifiable cyanobacterial contents (∼1–20%, or ∼105–106 copy numbers/g). Similar trends in 16S rDNA copy numbers were observed along the BSC vertical column structure (topsoil and subsurface).

For the BSC topsoils and subsurfaces, respective ordination analyses across the transect supported the presence of distinct bacterial communities, especially when considering the discrete grouping of BSCs from the high and low-density sites, as displayed in **Figures 5**, **6**. In summary, comparisons of sequences obtained through next generation methods showed substantial correlations with BSC surface coverage, including (A) changes at the phylum-level both across the transect and along the vertical column (e.g., increasing abundances of Chloroflexi and Cyanobacteria, respectively), (B) increasing diversity among the Cyanobacteria, (C) changes in distribution among the low-abundant genera (<15% relative abundance), (D) increasing OTU-level diversity in the BSC topsoils, (E) increases in OTU-level differentiation between the BSC topsoil and subsurface, and (F) increases in OTU-level differentiation between the BSCs and adjacent control soils. As listed in **Table 1**, the BSC topsoils were composed of Cyanobacteria (33 ± 8%), Proteobacteria (26 ± 6%), Chloroflexi (12 ± 4%), Actinobacteria (9.1 ± 1.3%), and Bacteroidetes (6 ± 2%); where the total sequences were numerically dominated by several OTUs from the *Phormidium* (Cyanobacteria) and an unidentified genus from Chloroflexi (AKIW781, order). At the phylum and OTU levels (**Tables 1**, **2**), the BSC subsurfaces were more diverse than the topsoils, and were primarily composed of Proteobacteria (23 ± 5%), Actinobacteria (20 ± 6%), and Chloroflexi (18 ± 3%); where the total sequences were numerically dominated by an unidentified genus from Chloroflexi (AKIW781, order) and *Rubrobacter* (Actinobacteria).

Across the transect, cyanobacterial abundances in the BSC topsoils were similar, which correlated with the trends in chlorophyll content. When expressed as relative cyanobacterial percentages, the dominating genera were *Phormidium* (47 ± 17%) and an unidentified genus from the *Xenococcaceae* family (19 ± 2%), with *Microcoleus* comprising only ∼1%, and those associated with the Nostocales order (e.g., *Nostoc*) representing ∼2% or less. In context, for BSCs from the northern Mojave Desert, Colorado Plateau, and Sonoran Desert, both cultivation-independent and dependent studies indicate that *Microcoleus* is the dominant cyanobacterial genus, irrespective of amplification using universal or cyanobacterial-specific primers (Redfield et al., 2002; Nagy et al., 2005; Rajeev et al., 2013). Additionally, cultivation studies on BSCs obtained near Edwards Air Force Base (in the western Mojave Desert) support *Microcoleus* as the dominant genus (Brostoff, 2002). Our results additionally diverge from other BSC studies in that diazotrophs from the Nostocales order (Cyanobacteria) comprise only a minor fraction of the total sequences, and that the best represented genera among this order are unidentified. In contrast, cultivation studies show that diazotrophs from Nostocales in BSCs from the Colorado Plateau are best represented by *Nostoc, Scytonema*, *Tolypothrix*, and *Spirirestis* (Yeager et al., 2004, 2007). However, using cultivation-independent methods, we show that these genera are <1% or undetectable in the BSCs from the central Mojave Desert. Thus, these next generation sequencing studies reveal a bacterial community profile that is distinct among BSCs found in the southwestern United States. These results additionally suggest that the key members involved in nitrogen fixation remain to be identified, which is important as correlations between C:N ratios (in the BSC topsoils) and increasing BSC surface density support an association between the flux of nitrogen fixation and BSC surface coverage.

Along the vertical structure of the BSCs, both two- and three-way statistical analyses supported the presence of unique communities in the BSC topsoils and subsurfaces. At the phylum-level, the BSC topsoils from the high-density site were enriched in cyanobacterial abundance and diversity, where, in contrast, the respective BSC subsurfaces were enriched in Actinobacteria and Acidobacteria at the high- and low-density sites, and in Nitrospirae at all sites. These distributions supported primary production in the BSC topsoil (as expected), and revealed vital roles for nitrogen and carbon metabolism in the BSC subsurface, as Nitrospirae play key roles in nitrification (Vos et al., 2011), and Actinobacteria are involved in biodegradation in soils (McCarthy and Williams, 1992).

In terms of metabolic activity, increases in BSC surface coverage also positively correlated with appreciable increases in intracellular ATP, catalase specific activities, and nitrogen contents (decreased C:N ratios). While ATP abundances are routinely used to estimate microbial abundance and activity (La Duc et al., 2004; Hammes et al., 2010; Dong and Zhao, 2015), our ATP measurements likely represent changes in metabolism of the BSCs across the transect. For instance, the measured changes in intracellular ATP are likely the result of fluxes in photosynthetic, aerobic, and anaerobic respiration, and not necessarily due to major changes in bacterial abundance or composition. In support are the 16S rDNA copy numbers, which were similar across the transect, and the myriad of phylogenetic changes which involved mostly lower percentage changes across the bacterial population. Hence, the ratiometric changes in intracellular ATP content (as expressed per gram or per 16S rDNA copy number) support a direct association between surface coverage and BSC metabolic activity. Furthermore, the appreciable amounts of extracellular ATP measured in the subsurface (at the low and intermediate-density sites) were mildly suggestive of biofilm formation within the BSC subsurface community (Xi and Wu, 2010; Rossi and De Philippis, 2015). Similarly, catalase specific activities (expressed per gram or 16S rDNA copy number) also positively correlated with surface coverage. For this study, catalase activities were used as a proxy for metabolic activity (inclusive of oxidative stress responses), as catalase degrades hydrogen peroxide, which is a by-product of photosynthesis (Apel and Hirt, 2004) and aerobic respiration (Cabiscol et al., 2010), and is additionally produced through photochemical reactions in desert soils (Georgiou et al., 2015) and water (Cooper and Zika, 1983; Yocis et al., 2000). As such, the ∼10-fold difference in catalase specific activities along the vertical column suggested that the BSC topsoils exhibit higher rates of respiration and/or experience higher levels of oxidative stress.

Increasing BSC surface coverages were also associated with several geochemical trends, including enrichments in clay and silt contents, and the biogenic elements of S, Ca, Mn, and Co, in the BSC topsoils. These trends suggested that the degree of BSC-mediated restructuring of the soil was associated with changes in both BSC metabolic activity and bacterial diversity. In contrast, as suggested by gradient analyses (**Figure 12A**), BSC surface coverages negatively correlated to broad increases in metal and metalloid (B, Ni, Mo, and Pb) abundances in the control soils; where, irrespective of trends, BSC surface coverages were diminished at sites with the highest abundances of B, Ca, Ti, Mn, Co, Ni, Mo, and Pb. In fact, in the BSC topsoils, the relative abundances of B, Ca, Cr, Ni, and Pb also negatively correlated with surface coverage; thereby, revealing a potential inhibitory role for heavy metals and/or boron across the transect.

Together, therefore, these results support the hypothesis that BSCs from regions of increasing surface coverage (along this transect) represent differing stages of early succession, which is known to involve changes in cyanobacterial morphology (Belnap and Lange, 2003), changes in cyanobacterial community structure (Zaady et al., 2010), increases in CO_2_ assimilation (Zaady et al., 2000), and increases in abundances of algae (Belnap and Lange, 2003). Gradient analyses (**Figure 12B**) bolster this assessment by supporting correlations between calculated BSC surface density and changes in bacterial community profiles (inclusive of cyanobacterial diversity) and increases in metabolism (intracellular ATP and catalase specific activities). Accordingly, our work expands upon the biogeography of BSCs and provides high resolution insights into the trends associated with early BSC succession in the Mojave Desert. Moreover, our findings suggest that BSC colonization and successional maturation are inhibited in the presence of appreciable metal/metalloid content, and reveal that the bacterial populations of BSCs in this section of the Mojave Desert, as measured through next generation sequencing, are unique among those found in the southwestern United States.

## Author Contributions

All authors contributed to the acquisition, analysis, or interpretation of the data, and drafting of critical revisions/reports of the work. All author’s approved the manuscript and agreed to be accountable for the work. The primary investigator and corresponding author is RM. Molecular genetics experiments were designed by PV, and primarily performed by PV, JCi, and EB. Bioinformatics analyses were performed by MBa. Surface density measurements were designed by KSc and EG, and imaging was performed by GC, RP, and PM. Project outcomes and data were reviewed by CM and RB. All members contributed to the biochemistry, soil chemistry, surface density, and PCR experiments/analyses, this included GPa, ST, JCa, JA, LB, DB, KB, MBr, MC, DC, GC, JCu, JM, CE, CG, RHa, RHo, SJ, BL, GM, EM, AO, GPe, NP, ER, VR, JR, MS, KSa, AS, GS, DS, JS, KSy, TRT, BW, and MW.

## Conflict of Interest Statement

The authors declare that the research was conducted in the absence of any commercial or financial relationships that could be construed as a potential conflict of interest.

## Footnotes

1 http://www.mothur.org/wiki/454_SOP

2 www.interactivenn.net/

## Supplementary Material

**FIGURE S1 |** Rarefaction and regression analysis of all BSC samples, where each sub-sample was analyzed in triplicate, and fits to the data were obtained through non-linear least squares regression using the standard form of a rectangular hyperpola, analagous to fitting with the Michaelis–Menten equation; data from each site were rarefied to 2020 OTU (high-density), 1780 OTU (intermediate-density), and 1770 OTU (low-density).

**FIGURE S2 |** Measurement of alpha-diversity by non-parametric comparison of the observed OTUs and Chao1 parameters through Monte Carlo simulations.
