# Supplementary figures and images for: Microbial Community and Biochemical Dynamics of Biological Soil Crusts across a Gradient of Surface Coverage in the Central Mojave Desert
Source: Front Microbiol. 2017 Oct 23;8:1974. doi: 10.3389/fmicb.2017.01974 (PMC5660283; doi:10.3389/fmicb.2017.01974)

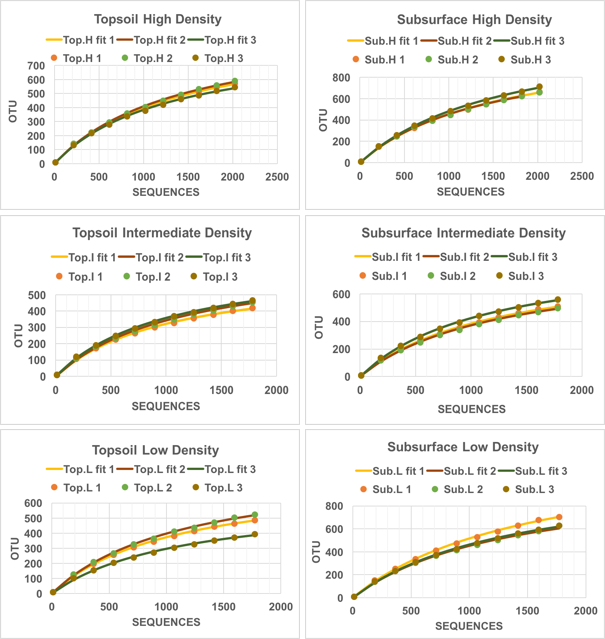

Supplement: Supplementary file 1 [file Image_1.PNG]

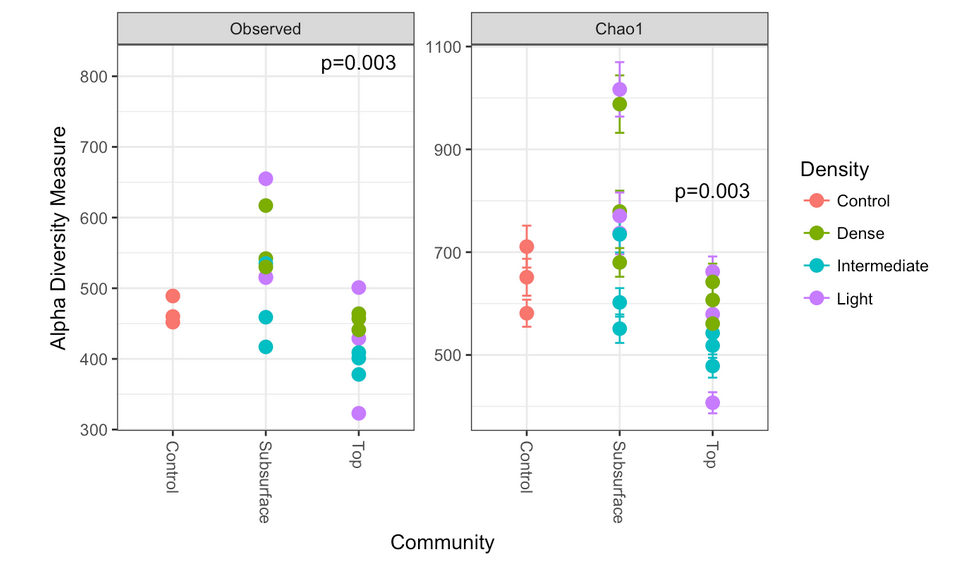

Supplement: Supplementary file 2 [file Image_2.PNG]
